# Genome-wide association identifies genomic regions influencing fillet color in Northwest Atlantic salmon (*Salmo salar* Linnaeus 1758)

**DOI:** 10.3389/fgene.2024.1402927

**Published:** 2024-07-26

**Authors:** Barbara L. Langille, Panya Sae-Lim, Solomon Boison, Philip G. Wiper, Amber F. Garber

**Affiliations:** ^1^ The Huntsman Marine Science Centre, Saint Andrews, NB, Canada; ^2^ Mowi Genetics AS, Bergen, Norway

**Keywords:** genome-wide association study, *bco1* gene, carotenoids, fillet color, selective breeding

## Abstract

Atlantic salmon (*Salmo salar*) is an important source of food globally; however, fillet color can significantly affect consumer purchasing, leading to potential food waste. Fish diets can be supplemented with astaxanthin to increase the organic pigment, carotenoid, responsible for flesh coloration; however, there is variation in the amount of overall fillet coloration in response to feeding astaxanthin. The uptake of this pigment is influenced by the environment and genetics and has been shown to be heritable. Therefore, we set out to determine the genomic associations of two separate year classes of farmed North American Atlantic salmon with measured Minolta Chroma Meter (lightness, redness, and yellowness) and SalmoFan phenotypic traits. Using ASReml-R genome-wide association, two genetic markers on chromosome 26 were significantly associated with almost all color traits, and these two markers explained between 6.0% and 12.5% of the variances. The genomic region on chromosome 26 was importantly found to be associated with the *beta-carotene oxygenase 1* (*bco1*) gene, which is essential in the conversion of beta-carotenoids to vitamin A, implying that this gene may also play an important role in flesh coloration in North American Atlantic salmon. Additionally, there were several genomic regions significantly associated with color traits, in which the accompanying genes had functions in line with thermogenesis, immune function, and pathogenic responses. Understanding how environmental and genetic factors work together to affect fillet quality traits will help inform genetic improvement.

## 1 Introduction

Selective breeding is an essential process behind sustainable aquaculture and has been used in large-scale production for decades as a way to keep up with the ever-growing demand for fish-based protein (Ahmed et al., 2022; Yáñez et al., 2023). Traditionally, breeding programs have relied on phenotypic information and pedigree crosses to select for fish (Zenger et al., 2017) or families of fish, with increasingly improved industry-conscious traits and fillet quality traits, where industry-conscious traits are crucial for the industry (e.g., faster growth rates and efficiency of feed) and fillet quality traits are essential for the consumer (e.g., fillet color and marbling) (Johnston et al., 2006). This form of selection has been widely employed in farmed species such as Atlantic salmon (*Salmo salar* Linnaeus 1758; e.g., Powell et al., 2008; Garber et al., 2019), coho salmon (*Oncorhynchus kisutch* Walbaum 1792; e.g., Neira et al., 2004), rainbow trout (*O. mykiss* Walbaum 1792; e.g., Haffray et al., 2012; Hu et al., 2013), Arctic charr (*Salvelinus alpinus* Linnaeus 1758; e.g., Wolters et al., 2013), and European whitefish (*Coregonus lavaretus* Linnaeus 1758; e.g., Kause et al., 2011; Quinton et al., 2007). However, once a robust baseline of phenotypic data has been collected, continual gathering can be inefficient, time-consuming, lethal, and wasteful of fish (Ibtisham et al., 2017). To date, an increasing number of trait-specific genomic markers have been reported (see Table 6 in Yáñez et al., 2023), and some studies have shown improved prediction accuracy compared to pedigree methods (e.g., Tsai et al., 2015; Bangera et al., 2017; Correa et al., 2017; Horn et al., 2020).

The economically valuable red/yellow coloration of Atlantic salmon fillets, an important fillet quality trait, is caused by the binding of unmetabolized carotenoid pigments (i.e., the pigment that does not get metabolized in the liver or enterocytes) to muscle alpha-actinin (Castenmiller and West, 1998; Matthews et al., 2006). Astaxanthin, one of the most common carotenoid pigments, is commonly supplemented in fish diets and has been estimated to account for 10%–15% of feed costs (Norris and Cunningham, 2004). Therefore, understanding its influence on muscle pigmentation is important from a cost perspective. Studies on muscle coloration have revealed that fish with supplemented astaxanthin have an overall redder coloration than those without (Thomas, 1999; Arredondo-Figueroa et al., 2007), indicating that diet has the ability to significantly impact color retention in muscle. Despite the general success of supplementing diets with astaxanthin, a more recent study discovered significant differences in color retention between different strains of rainbow trout, in which fish were given the same non-pigment-enhanced diet (Crouse et al., 2018), implying there were other factors aside from diet influencing the uptake of pigment into muscle. Additionally, standard pigment supplementation does not translate into the same level of muscle coloration for all fish. Therefore, the intensity of muscle coloration can be influenced by a variety of situations such as handling and storage conditions (Colihueque, 2010) but most notably diet and genetics (Gjerde and Gjedrem, 1984; Rye and Gjerde, 1996; Neira et al., 2004; Garber et al., 2019).

Genomic and functional studies have been able to link variation in muscle coloration to beta-carotene 15,15′-oxygenase (BCO1). In mammals, the BCO1 enzyme is important in the conversion of beta-carotene to retinal (a form of vitamin A) and has been found to be relatively functionally conserved in a variety of mammal and non-mammal species, including humans (Ferrucci et al., 2009; Leung et al., 2009; Lietz et al., 2012; Yabuta et al., 2016), chickens (Le Bihan-Duval et al., 2011), mice (Hessel et al., 2007; Amengual et al., 2020), and fish (Helgeland et al., 2019; Blay et al., 2021; Ahmed et al., 2022; Sae-Lim et al., 2022). A recent study on European Atlantic salmon found functional links between redness muscle coloration and the *beta-carotene oxygenase 1 like* (*bco1*) gene (Helgeland et al., 2019), which is likely the most important gene involved in the redness coloration in salmon. A follow-up study by Sae-Lim et al. (2022) also identified this same region on chromosome ssa26 in Atlantic salmon from Norway, although they also found an additional region on chromosome ssa2 (encompassed the inorganic phosphate—*ppaib* gene) that was significantly associated with redness coloration. However, North American Atlantic salmon has undergone chromosomal rearrangements from its counterpart in Europe (Gao et al., 2023), and studies have shown salmon from either side of the Atlantic to be genomically distinct (King et al., 2001; 2007; Verspoor et al., 2005). Therefore, it is possible that different or additional regions could be associated with flesh coloration in North American Atlantic salmon.

Redness has been identified as an important trait in the coloration of salmon; however, there are other color-related traits within muscle, such as yellowness and lightness, which contribute to the overall appearance of the fillet (Moroney et al., 2014). A genome-wide association study (GWAS) on rainbow trout utilizing redness, yellowness, and lightness measurements found distinct peaks on different chromosomes for each trait. Authors were able to link these peaks to a variety of genes with functions involving carotenoid metabolism and myoglobin homeostasis (Ahmed et al., 2022), adding to the growing body of evidence supporting carotenoid involvement in not only pigmentation but also in oxidative stress as well (Nakano and Wiegertjes, 2020 and references therein). Aquaculture production environments can involve changing temperatures and salinity, use of chemicals, different lighting, and altered diets, which can all create an imbalance between the production of reactive oxygen species (ROS) and the antioxidant defense system in fish (Martinez-Alvarez et al., 2005), often leading to an increased risk of disease (see Nakano and Wiegertjes, 2020 for a review). Dietary carotenoids have been shown to improve antioxidative enzymes for improved disease defense and also strengthen general resistance to oxidative stress (Nakano and Wiegertjes, 2020). Aside from the role that carotenoids play in red/yellow muscle pigmentation and oxidative stress, they have also been linked to growth, survival, and immune responses (Tacon, 1981; Alishahi et al., 2015; Cheng et al., 2018; Abd El-Gawad et al., 2019; Liu et al., 2019). Therefore, there is potential for the GWAS to identify regions of the genome that may be correlated with pigment and involved in other aspects of fish health/quality.

There are two common ways to measure color in fillets: using a Chroma Meter and a SalmoFan. A Chroma Meter is an instrument that objectively measures color, while a SalmoFan is a color fan where blades are arranged in an ordered gradient of color (i.e., from pale pink/peach to a deep red/yellow for salmonids), which is used to visually assess fillet colors. However, the SalmoFan score depends on the color-perceiving ability of the person who measures fillet color and, therefore, may be subjected to bias. Despite potential biases, measurements taken by a SalmoFan have been shown to be heritable and can also be connected to specific quantitative trait loci in salmon (Baranski et al., 2010). Yet, it is still unknown whether SalmoFan measurement provides similar results to Chroma Meter color traits, given its potential to provide bias from: being used by different people, different times of the day, and different levels of fatigue.

Here, we aim to use genome-wide association studies in 2 year classes (YC) of farmed North American-origin Atlantic salmon to identify regions of the genome associated with important fillet color traits. Specifically, we set out to 1) identify the regions of the genome significantly associated with lightness, chromaticity (redness and yellowness), and SalmoFan measurements; 2) determine a genomic overlap between different traits; and 3) evaluate if the subjective SalmoFan measurement provides similar genetic parameters and genome-wide association to the Chroma Meter color traits.

## 2 Methods

### 2.1 Breeding program

A breeding program was started by the Huntsman Marine Science Center (Huntsman, St. Andrews, New Brunswick, Canada) in 2010 (now managed with Mowi Canada East, MCE). The first pedigreed YC was produced in 2010 using a partial factorial mating design from previously domesticated broodstock. Similarly, the second pedigreed YC was produced in 2011. These original broodstock had been maintained by Northern Harvest Sea Farms (NHSF, purchased by MCE). Selected individuals from the 2010 YC primarily produced the 2014 YC and, subsequently, the 2018 YC. Selected individuals from the 2011 YC primarily produced the 2015 YC and, subsequently, the 2019 YC with minimal contribution of 2010 or 2014 as 5-year-olds to 2015 and 2019. However, in the production of the 2014 YC, there was a contribution from previously unpedigreed Atlantic salmon from Dover fish farms (purchased by NHSF). A microsatellite marker multiplex and/or low-density SNP array were used to estimate relatedness prior to pedigree construction to increase the initial genetic variability and avoid potential in breeding. The 2018 and 2019 crosses were made in Prince Edward Island, Canada (MCE). The 2018 crosses were transferred to individual family tanks at the Huntsman and reared to an average weight of −0.8 g, at which time a commercial group was created, transferred to a commercial hatchery, and subsequently to a commercial sea cage site in New Brunswick, Canada. Since the fish from the 2018 YC were not retained in separate family tanks for very long, we expect the common environmental effect due to family tanks to be insignificant in this case. The 2019 crosses were mixed in equal numbers as eyed eggs and then transferred to a commercial hatchery, followed by a commercial sea cage site (similar to 2018). Appropriately sized diets (Skretting, Canada) were fed to each life stage of the salmon using their recommended diet tables for commercial hatchery and sea cage production (feed contained added astaxanthin with unknown inclusion levels). Feeding and husbandry regimes were similar across the production of both year classes. Entire sea cages were harvested at −10,900° days (−1175 days post-fertilization or dpf) for the 2018 YC and −10,350° days (−1175 dpf) for the 2019 YC. A random subset totaling 1,669 individuals were selected from the 2018 YC by removing the first seven salmon from tubs, in which fish were being held during the harvest. A random subset totaling 1,955 individuals were selected from the 2019 YC by removing one salmon every −30 s from a conveyor belt. Salmon in 2018 were transported into the plant in tubs, whereas in 2019, they were transported in tanker trucks.

Detailed carcass traits, such as weight, sex, and maturity, were recorded alongside quality traits, such as melanin discoloration, gaping, and marbling (see Garber et al., 2019 for further description of traits recorded during harvest evaluation). Fillet color traits were recorded using a SalmoFan™ (Roche, 1997-HMB, Switzerland) and a Minolta Chroma Meter (CR-410, C illuminate). The Chroma Meter recorded lightness (L* which goes from white to black) and chromaticity (redness or green to red—a* and yellowness or blue to yellow—b*). L*, a*, and b* measurements were recorded above the midline centered between the start of the dorsal fin and the lateral line and below the midline directly below the first measurement centered between the lateral line and ventral surface of the fillet. To remove any potential variability caused by equipment operation, the same individual ran the Chroma Meter each day. Information from the Chroma Meter was fed directly into a computer which was in a separate location. Therefore, the SalmoFan operator could not be biased based on the values from the Chroma Meter. One individual (different from the Chroma Meter operator) was responsible for all SalmoFan measurements, which took place in a light box to increase uniformity across the day with no change in internal room lighting during the assessment. The SalmoFan measurements were in the same location as the above-the-midline Chroma Meter measurement. All fillet color data were recorded from the right fillet only. Finally, fin clips were collected and stored in 2-mL Eppendorf tubes filled with 95% ethanol, which were initially sent for genetic analysis to create the pedigree (parentage assignment and relatedness to determine siblings) of the assessed individuals during the harvest evaluation.

### 2.2 Phenotypic data and pedigree variance component estimates

Both harvest evaluations took place in the same month (January 2022 and January 2023); however, the 2019 YC had 124 fully mature fish, while the 2018 YC did not. In order to have comparable YC, these mature fish were removed as they could have a higher body weight or a different concentration of color in fillets, solely based on being in a different life stage. All the individuals with a SalmoFan of 10 (i.e., almost white in color) were mature; therefore, they were all removed when the mature individuals were filtered out. Previously, Langille et al. (in review) revealed that timing factors (i.e., the day of the harvest, the hour of the day, and the “duration,” which referred to the amount of time between the first measurement upon entering the processing facility to the final color measurement) had significant effects on fillet color traits in eight YCs. In the 2018 and 2019 YC, there were two and three harvests of fish, respectively, and the duration differences between fish at its maximum were 10.7 h in 2018 and 15.0 h in 2019. The attached magnitude of the effect table for the significance of timing effects on each trait is shown in Supplementary Table S1. Therefore, all these fixed effects were incorporated in the statistical analysis. The phenotypic traits (L*, a*, and b*; SalmoFan) of each YC were evaluated separately by fitting the below univariate animal mixed model in ASReml-R 4.1 [*asreml* flag in *asreml* package; Butler et al. (2009)] in R version 4.3.0 (R Development Core Team. (2015)):

$$y_{ijklmno}=\mu +{\text{sex}}_{i}+{\text{mature}}_{j}+{\text{day}}_{k}+{{\text{time}}_{l}+\text{hour}}_{m}+\beta {\text{weight}}_{n}+a_{o}+e_{ijklmno},$$

where y is the data on the phenotypic measurements; μ is the overall mean; ${\text{sex}}_{i}$ is the fixed effect of observations (*i* = 1: male, 2: female, and 3: unknown); ${\text{mature}}_{j}$ is the fixed effect corrected for maturity (*j* = 1: immature fish—thread-like gonads and 2: fish that are undergoing maturation and will spawn later in the same year as assessment–gonads are beginning to develop); and ${\text{day}}_{k}$ , ${\text{time}}_{l}$ , and ${\text{hour}}_{m}$ are the fixed effect of timing factors corrected for the *k*th day of harvest, for the *l*th time taken to process an individual fish, and for the *m*th hour of the day, respectively. ${\text{weight}}_{n}$ is the fixed covariate included in the model to account for the relationship between phenotype traits and body weight of the *n*th individual, where β is the regression coefficient. $a_{o}$ is the random additive genetic effect, a ∼ *N* (0, **A**σ_*a*_^2^), of the *o*th animal, where **A** is the numerator relationship matrix and $e_{ijklmn}$ is the random residual effect; ***e*** ∼ *N* (0, **I**σ_*e*_^2^), where **I** is the identity matrix. All fixed effects were statistically tested using the Wald test with the *wald.asreml* flag. Narrow-sense heritabilities were estimated using *vpredict* in the ASReml package as $h^{2}=\sigma_{a}^{2}/\left(\sigma_{a}^{2}+\sigma_{e}^{2}\right)$ , where $\sigma_{a}^{2}$ and $\sigma_{e}^{2}$ are the genetic and residual variances, respectively. In 2019, there were 19 fish that had a physical watermark from being in frozen water at the bottom of the tub, which could have either affected the Chroma Meter or the SalmoFan measurement; thus, watermarked fish were removed.

The function *pairs.panels* in the *psych* package (in R; Revelle, 2023) was utilized to estimate phenotypic correlations among bled weight, gonad weight, condition factor, and color traits. We chose to use bled weight; however, bled weight was also highly correlated with fork length, carcass weight, and fillet weight (>0.90); therefore, any one of these traits could be used. *Pairs.panels* creates a data matrix with Pearson correlations above the diagonal, bivariate scatterplots below the diagonal, and histograms on the diagonal.

All traits (color traits and bled weight) were re-assessed using a bivariate animal mixed model to estimate the covariance components in ASReml-R:

$$\left[\begin{array}{c} y_{1}\\ y_{2} \end{array}\right]=\left[\begin{array}{cc} X_{1} & 0\\ 0 & X_{2} \end{array}\right]\left[\begin{array}{c} b_{1}\\ b_{2} \end{array}\right]+\left[\begin{array}{cc} Z_{1} & 0\\ 0 & Z_{2} \end{array}\right]\left[\begin{array}{c} a_{1}\\ a_{2} \end{array}\right]+\left[\begin{array}{c} e_{1}\\ e_{2} \end{array}\right],$$

where $y_{1}$ and $y_{2}$ are the data vectors of the phenotypic measurements of interest; $X_{1}$ and $X_{2}$ are the incidence matrices of the fixed effects and covariate as described in the univariate animal mixed model above (bled weight was added as the covariate when color traits were assessed), respectively; $b_{1}$ and $b_{2}$ are the solution vectors for corresponding fixed effects; $Z_{1}$ and $Z_{2}$ are the incidence matrices of the random animal effects; $a_{1}$ and $a_{2}$ are additive genetic effects for the first and second phenotypes following multivariate normal distribution (*MVN*) for the pedigree relationship matrix (**A**): $\left[\begin{array}{c} a_{1}\\ a_{2} \end{array}\right]∼MVN\left(\left[\begin{array}{c} 0\\ 0 \end{array}\right],\left[\begin{array}{cc} \sigma_{a_{1}}^{2} & \sigma_{a_{1},a_{2}}\\ \sigma_{a_{1},a_{2}} & \sigma_{a_{2}}^{2} \end{array}\right]⊗A\right)$ ; $e_{1}$ and $e_{2}$ are the solution vectors of random residuals following normal distribution: $\left[\begin{array}{c} e_{1}\\ e_{2} \end{array}\right]∼MVN\left(\left[\begin{array}{c} 0\\ 0 \end{array}\right],\left[\begin{array}{cc} \sigma_{e_{1}}^{2} & \sigma_{e_{1},e_{2}}\\ \sigma_{e_{1},e_{2}} & \sigma_{e_{2}}^{2} \end{array}\right]⊗I\right)$ . The *summary* flag was used to view the variance components output by the model, all in ASReml-R.

### 2.3 Genotypic data—quality control and GWAS preprocessing

Fin clips were sent to IdentiGEN for DNA extraction through genotyping using a Thermo Fisher Axiom genotyping array containing 55,725 single-nucleotide polymorphism (SNP) markers distributed evenly across the 27 chromosomes of the North American Atlantic salmon genome (the 50K Axiom SNP array; fully described by Gao et al., 2023).

Standard quality control filtering was performed in PLINK v1.9 (Purcell et al., 2007; http://pngu.mgh.harvard.edu/purcell/plink/) according to the following criteria: retained markers with minor allele frequency (MAF) ≥ 0.05 (--*maf* flag; Supplementary Figure S1), removed markers and individuals with >10% missing data (--*geno* and --*mind* flags, respectively), and removed individuals that had pi-hat values over 0.9 as they were likely duplicates (--*genome* flag). Finally, the *adegenet* (Jombart, 2008) and *hierfstat* (Goudet, 2004) packages were used to measure genetic diversity, including observed and expected heterozygosities (*H*_*O*_ and *H*_*E*_) and inbreeding (*F*_*IS*_).

As there is a family structure and, therefore, non-random individual relationships, the large-scale population structure was evaluated by principal component analysis (PCA). We used the R package *pcadapt* (Luu et al., 2017) with an initial cluster (*K*) value of 50 and *min.maf* of 0.01. We determined the markers that were significantly correlated with the PCA (loci with *q-*values < 0.05) as they could be loci that were influenced by artificial mating selection. However, there was no change in the eigenvectors when computed with or without those significant markers (12 markers in total). The *plot* function in R was used to visualize the data. To evaluate the small-scale population structure, a kinship matrix was generated using the *gVanRaden.2* function in the *gmatrix* package in R. This package utilizes the SNP information to generate genomic relatedness. The *heatmap.2* function in *gplot* was used to visualize the data.

### 2.4 Genomic variance component estimate and genome-wide association

The package *ASRgwas* is a linear mixed-model approach, which is used in tandem with *ASReml-R* to estimate genomic variance components and run the GWAS analysis. The same linear mixed model used for the preliminary pedigree variance component estimates was used to estimate genomic variance components, except that the numerator relationship matrix (**A**) was replaced with a genomic relationship matrix (**G**) constructed using (VanRaden, 2008). To run GWAS analysis, two additional parameters were added to the variance component estimation model. We fitted the regression of eigenvectors and the marker effect for each trait using the function *gwas.asreml*. The number of PC (*npc*) axes was included in the analysis and determined by a scree plot that was generated from the PCA obtained in the previous section (five axes for the 2018 YC and six axes for the 2019 YC). The *p-*value threshold (*p-value.thr*) for significant loci was set to 5.0e-6 and lowered until the false discovery rate was under 5% (evaluated at the end of each run). Quantile–quantile (QQ) and Manhattan plots were generated using the *qqplot* and *manhattan* functions of the *qqman* package in R (Turner, 2014).

### 2.5 Gene region discovery

Once the gene regions of interest were determined from the GWAS, rapid Ensembl v.109 (Cunningham et al., 2022) and the North American Atlantic salmon assembly (*S. salar* USDA_NASsal_1.1, INSDC Assembly GCA 021399835.1; https://rapid.ensembl.org/Salmo_salar_GCA_021399835.1/Info/Index?db=core;r=26:42047177-42067177) were utilized to find all associated genes (including a 50K region on either side of outlier SNPs). The Ensembl gene lists were then run in Metascape (http://metascape.org) (Zhou et al., 2019) to generate a list of overlapping Gene Ontology (GO) terms.

## 3 Results

### 3.1 Phenotypic variance, correlations, and heritability

All individuals were assigned back to 83 families (48 sires and 57 dams) and 207 families (102 sires and 125 dams) from the 2018 and 2019 YC, respectively. There were between 1 and 34 individuals per family with the mean/median being 12.87/13 in the 2018 YC and 7.64/7 in the 2019 YC.

**FIGURE 1:**
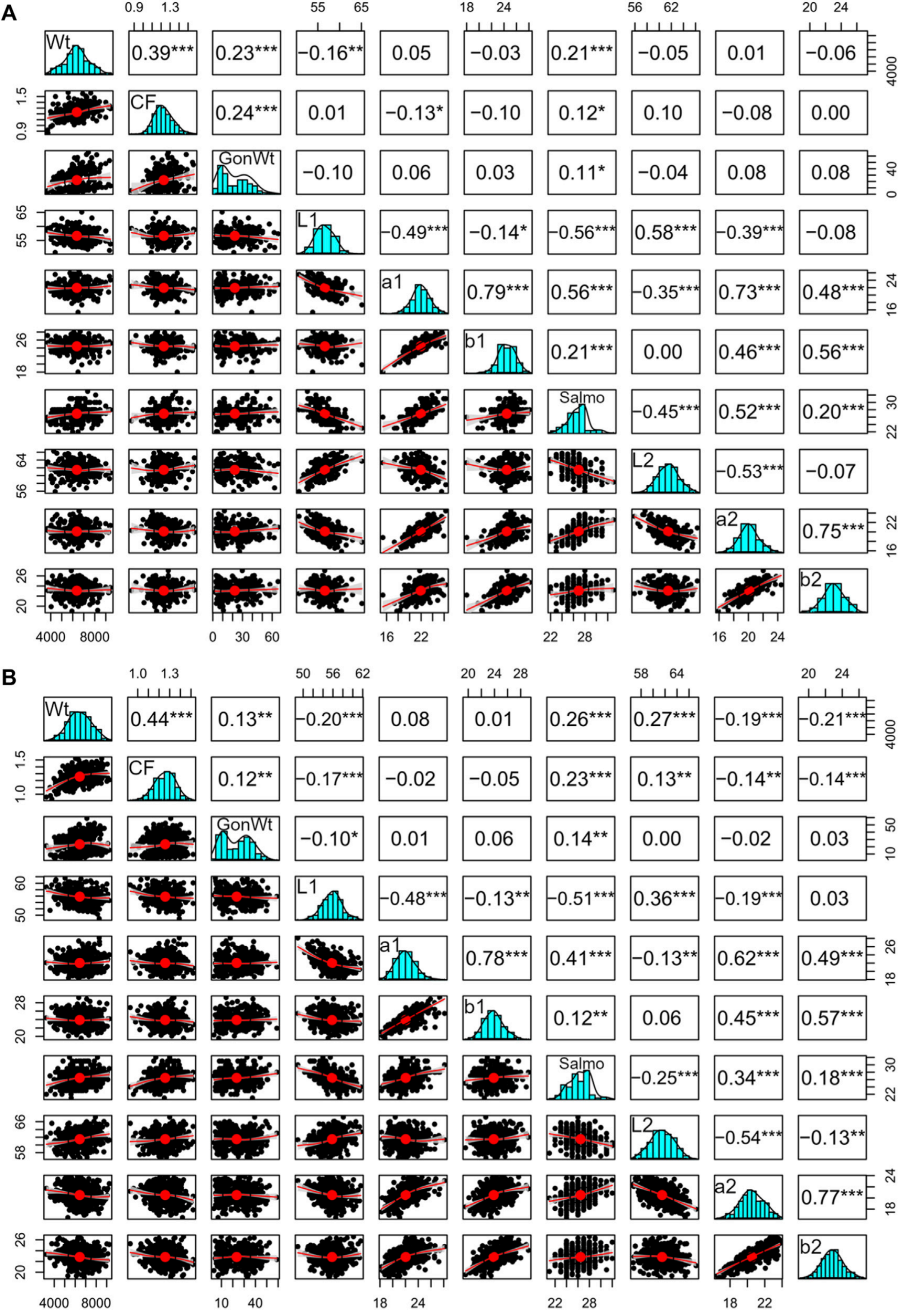
Correlated phenotypic variables in the **(A)** 2018 and **(B)** 2019 YC Atlantic salmon that could influence the expression of fillet color traits, where values above the diagonal are Pearson correlations, bivariate plots are below the diagonal, and histograms are on the diagonal. “Wt” is the bled weight, “CF” is the condition factor, “GonWt” is the gonad weight, and “Salmo” is the SalmoFan. Asterisk (*) refers to the confidence interval where * is a *p-value* < 0.05, ** is a *p-value* < 0.01, and *** is a *p-value* < 0.001.

In general, the fillets were lighter and with much less coloration below the midline. a* (redness) and b* (yellowness) above the midline were between 1.59 and 2.21 and 1.04 and 1.48 higher than below the midline, respectively, based on differences between means (Table 1). We found that color traits from above and below the midline were phenotypically correlated (i.e., L*1 and L*2, a*1 and a*2, and b*1 and b*2), although a* and b* had a slightly higher correlation than the L* measurements (Figure 1). The phenotypic correlation among the color traits (not between above and below) was low to moderate. The a* and b* measurements were positively correlated, while the L* measurement was generally negatively correlated to the a* and the b* measurements. The negative correlation of lightness to the other color traits indicates that as fish became more red/yellow, they were generally darker. In addition, the phenotypic correlations of SalmoFan to a* and b* were lowly positive and moderately negative to L*. Finally, all the color traits were weakly correlated with the size of the fish with values ranging from −0.21 to 0.27.

**TABLE 1:** Descriptive statistics of color-related phenotypes from both year classes. Pedigree and genomic heritability (*h* ^2^) estimates and their associated standard errors (SEs). Traits ending in “1” were measured above the midline, while traits ending in “2” were measured below the midline.

Year	Trait	Mean	SD	Min	Max	*Pedigree h* ^2^ ± SE	*Genomic h* ^2^ ± SE
2018	Lightness (L*1)	55.72	2.00	49.23	65.11	0.21 ± 0.06	0.36 ± 0.05
	Redness (a*1)	21.65	1.58	15.36	26.68	0.51 ± 0.09	0.54 ± 0.05
	Yellowness (b*1)	23.82	1.46	18.01	29.58	0.32 ± 0.07	0.38 ± 0.05
	SalmoFan1	26.50	1.74	21.00	33.00	0.46 ± 0.08	0.40 ± 0.05
	Lightness (L*2)	61.42	1.70	54.84	67.79	0.27 ± 0.07	0.43 ± 0.05
	Redness (a*2)	20.06	1.44	15.39	24.46	0.55 ± 0.09	0.53 ± 0.05
	Yellowness (b*2)	22.78	1.39	17.71	29.01	0.39 ± 0.08	0.42 ± 0.05
2019	Lightness (L*1)	54.38	2.31	48.00	62.52	0.30 ± 0.05	0.36 ± 0.05
	Redness (a*1)	22.87	1.79	17.63	28.12	0.48 ± 0.06	0.49 ± 0.05
	Yellowness (b*1)	24.54	1.64	19.00	30.66	0.45 ± 0.06	0.43 ± 0.05
	SalmoFan1	26.51	1.80	20.00	32.00	0.43 ± 0.06	0.45 ± 0.05
	Lightness (L*2)	61.80	1.89	55.89	68.38	0.30 ± 0.06	0.37 ± 0.05
	Redness (a*2)	20.56	1.45	16.10	25.56	0.59 ± 0.07	0.55 ± 0.04
	Yellowness (b*2)	23.08	1.46	17.53	28.40	0.52 ± 0.07	0.47 ± 0.05

Abbreviations correspond to heritability (*h* ^2^), standard deviation (SD), and standard error (SE).

The pedigree-based heritability of lightness was the lowest among the color traits in both YC (between 0.21 and 0.30), while the heritability of redness, yellowness, and SalmoFan was consistently higher ranging from 0.32 (2018 yellowness) to 0.59 (2019 redness) (Table 1). The genetic correlations between the color traits for the measurement above and below the midline were moderate to highly correlated (0.615–0.879 for L*, 0.933 to 0.983 for a*, and 0.873 to 0.925 for b*) for both YC (Table 2). Furthermore, the genetic correlation between SalmoFan and the other color traits were moderately to highly positive (a* and b* measurements) but moderately negative to the L* measurements. Genetically, some of the color traits were weakly positive (a* and SalmoFan) to weakly negatively (L*) correlated with bled weight (ranged from −0.348 to 0.450) (Table 2), indicating that as weight increases, fillet color increases.

**TABLE 2:** Genetic correlations based on the bivariate model between all color traits and with bled weight. For the color traits, values above and below the diagonal are for the 2018 YC and 2019 YC, respectively. Traits ending in “1” were measured above the midline, while traits ending in “2” were measured below the midline. Z-ratios were used for the assessment of significance, where a value over 2 or under −2 was considered significant (indicated by yellow highlight in the table).

Trait	Bled weight		Color trait						
	18 bled weight	19 bled weight	L*1	a*1	b*1	SalmoFan1	L*2	a*2	b*2
L*1	−0.335 ± 0.194	−0.256 ± 0.122	NA	−0.812 ± 0.081	−0.448 ± 0.172	−0.954 ± 0.040	0.879 ± 0.068	−0.854 ± 0.078	−0.478 ± 0.171
a*1	0.394 ± 0.165	0.284 ± 0.114	−0.577 ± 0.092	NA	0.855 ± 0.050	0.886 ± 0.046	−0.609 ± 0.123	0.983 ± 0.015	0.777 ± 0.078
b*1	0.259 ± 0.193	0.169 ± 0.122	−0.104 ± 0.136	0.898 ± 0.028	NA	0.556 ± 0.131	−0.167 ± 0.190	0.852 ± 0.061	0.925 ± 0.042
SalmoFan1	0.450 ± 0.156	0.138 ± 0.128	−0.887 ± 0.042	0.696 ± 0.078	0.410 ± 0.114	NA	−0.888 ± 0.060	0.912 ± 0.041	0.521 ± 0.133
L*2	−0.049 ± 0.207	−0.348 ± 0.119	0.615 ± 0.108	−0.469 ± 0.107	−0.329 ± 0.120	−0.800 ± 0.068	NA	−0.655 ± 0.109	−0.244 ± 0.184
a*2	0.322 ± 0.178	0.029 ± 0.119	−0.609 ± 0.090	0.933 ± 0.025	0.843 ± 0.046	0.773 ± 0.065	−0.693 ± 0.070	NA	0.841 ± 0.052
b*2	0.232 ± 0.197	0.057 ± 0.121	−0.497 ± 0.108	0.827 ± 0.048	0.873 ± 0.037	0.613 ± 0.089	−0.522 ± 0.101	0.906 ± 0.026	NA

### 3.2 Genomic data and population stratification

There were a total of 35,241 and 34,966 SNPs in the 2018 YC and 2019 YC, respectively, after quality control filtering. The 2018 YC had an average *H*_*O*_ of 0.427 and a highly significant Bartlett’s k-squared value of 213.21 (*p-*value = 2.2e-16). The 2019 YC had an average *H*_*O*_ of 0.421 and a highly significant Bartlett’s k-squared value of 55.15 (*p-*value = 1.11e-15), indicating that inbreeding is likely playing a role in the structuring of fish from both years (2018 *F*_*IS*_ = −0.246; 2019 *F*_*IS*_ = −0.327). The values of *F*_*IS*_ were large and negative, which could be due to the allele frequency and an excess of heterozygosity relative to what would be expected. The observed heterozygosity was higher than expected, likely due to polymorphic markers, validating the *F*_*IS*_ result.

**FIGURE 2:**
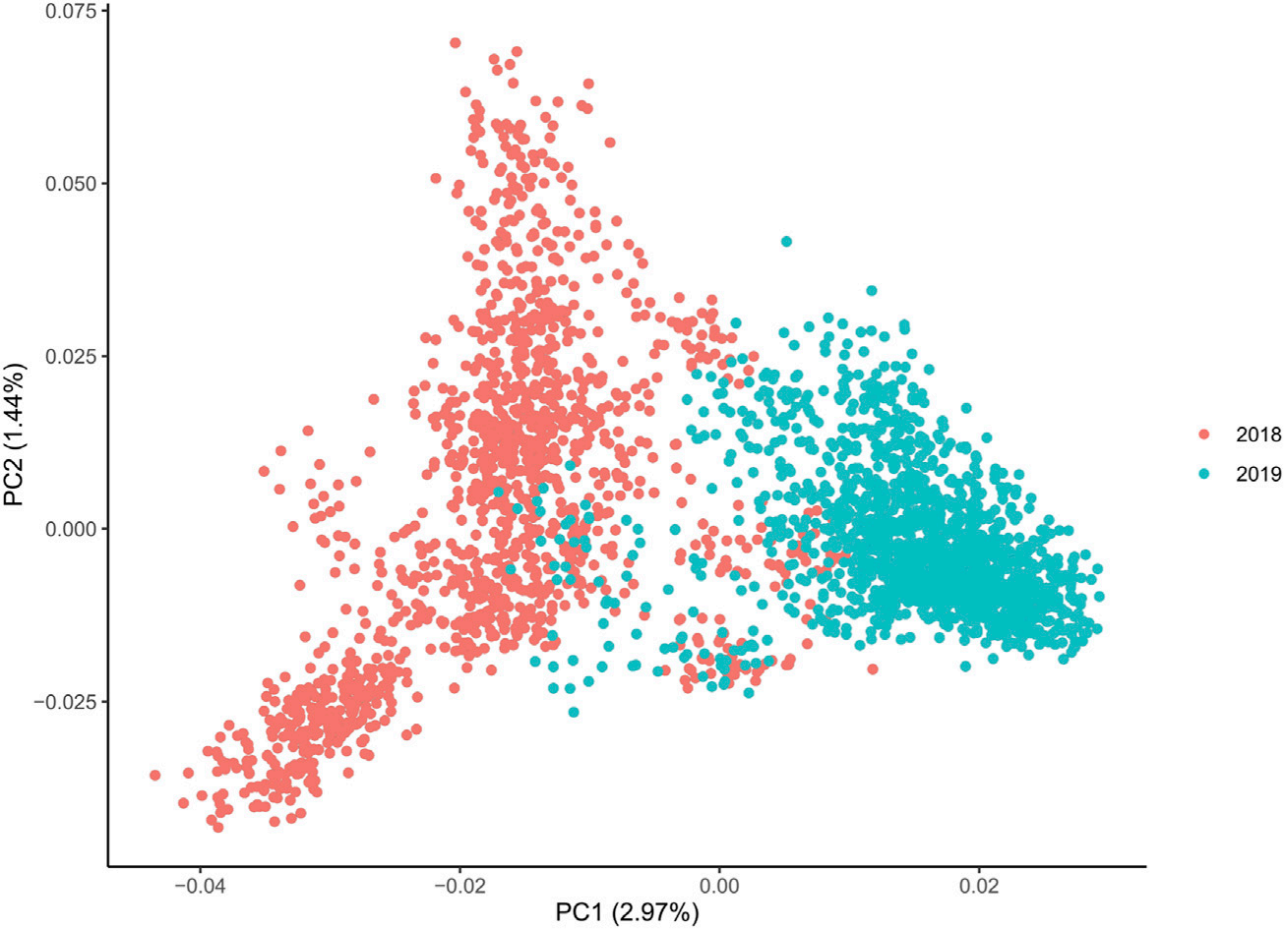
Principal component analysis of both year classes, where the optimal number of components is 4 based on the scree plot, and all axes display the same level of differentiation between 2018 and 2019 YC.

**FIGURE 3:**
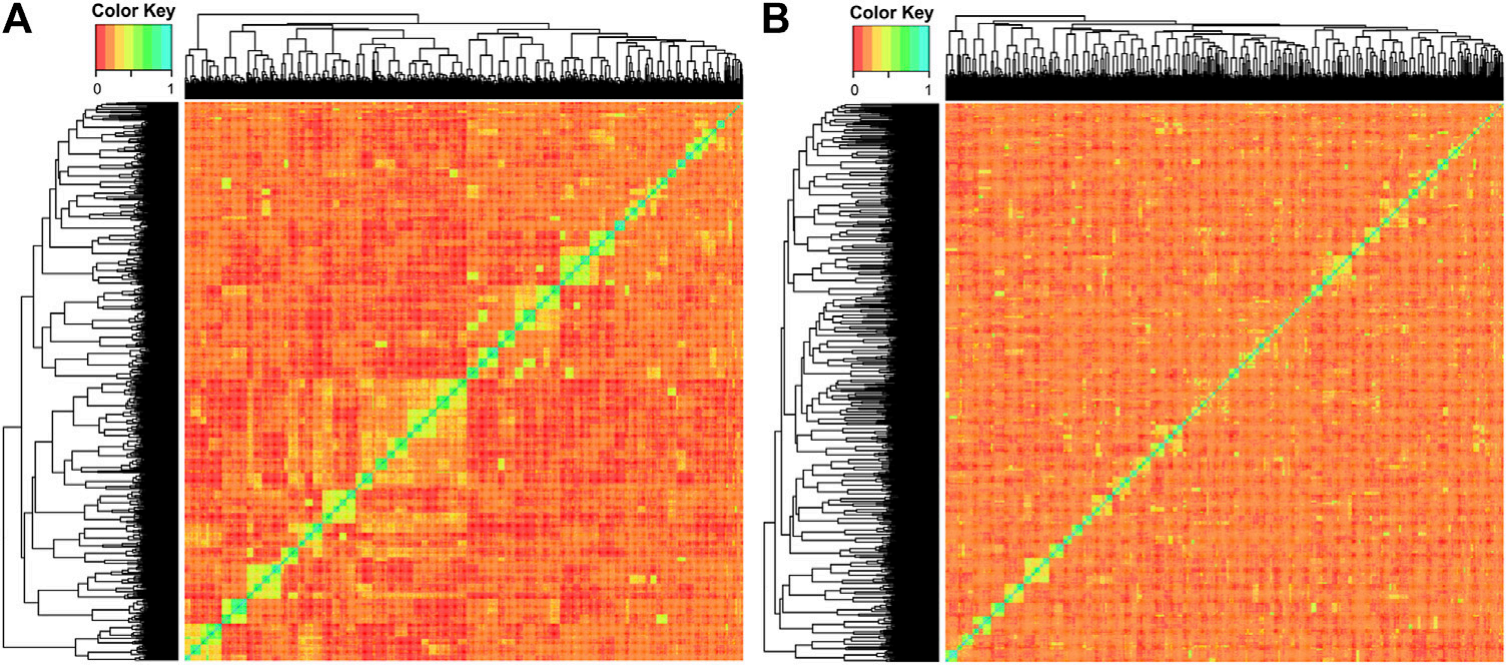
Kinship matrix of **(A)** 2018 YC and **(B)** 2019 YC, where higher relatedness is visualized by the color scale bar, where red is 0 and cyan is 1.

The PCAs split both YC along the primary axis (2.97% of the explained variance; Figure 2). The 2018 and 2019 YC originated primarily from different founder fish; therefore, the separation between years is not surprising. Interestingly, the 2019 YC was an overall tighter group, while the 2018 YC had further population-level division within, also along the primary PC axis. Despite lower overall inbreeding, the 2018 YC could have a few larger groups consisting of more highly related individuals than those in the 2019 YC, which could be driving much of the within group division. Indeed, the kinship matrix was able to identify highly related individuals in both YC (Figure 3); however, the smaller number of families (and more individuals per family) in the 2018 YC were likely contributing to the pattern of larger-looking pockets of relatedness. Therefore, correcting for kinship was important in the GWAS analysis.

### 3.3 Genomic heritability and genome-wide association

Genomic *h* ^2^ was generally higher than pedigree-based *h* ^2^ in the 2018 YC and slightly lower in the 2019 YC (Table 1). The largest difference in the *h* ^2^ estimates between the pedigree and genomic information was for lightness in the 2018 YC (0.21 vs. 0.36). Lastly, the standard errors for both genomic and pedigree *h* ^2^ estimates were generally low.

**FIGURE 4:**
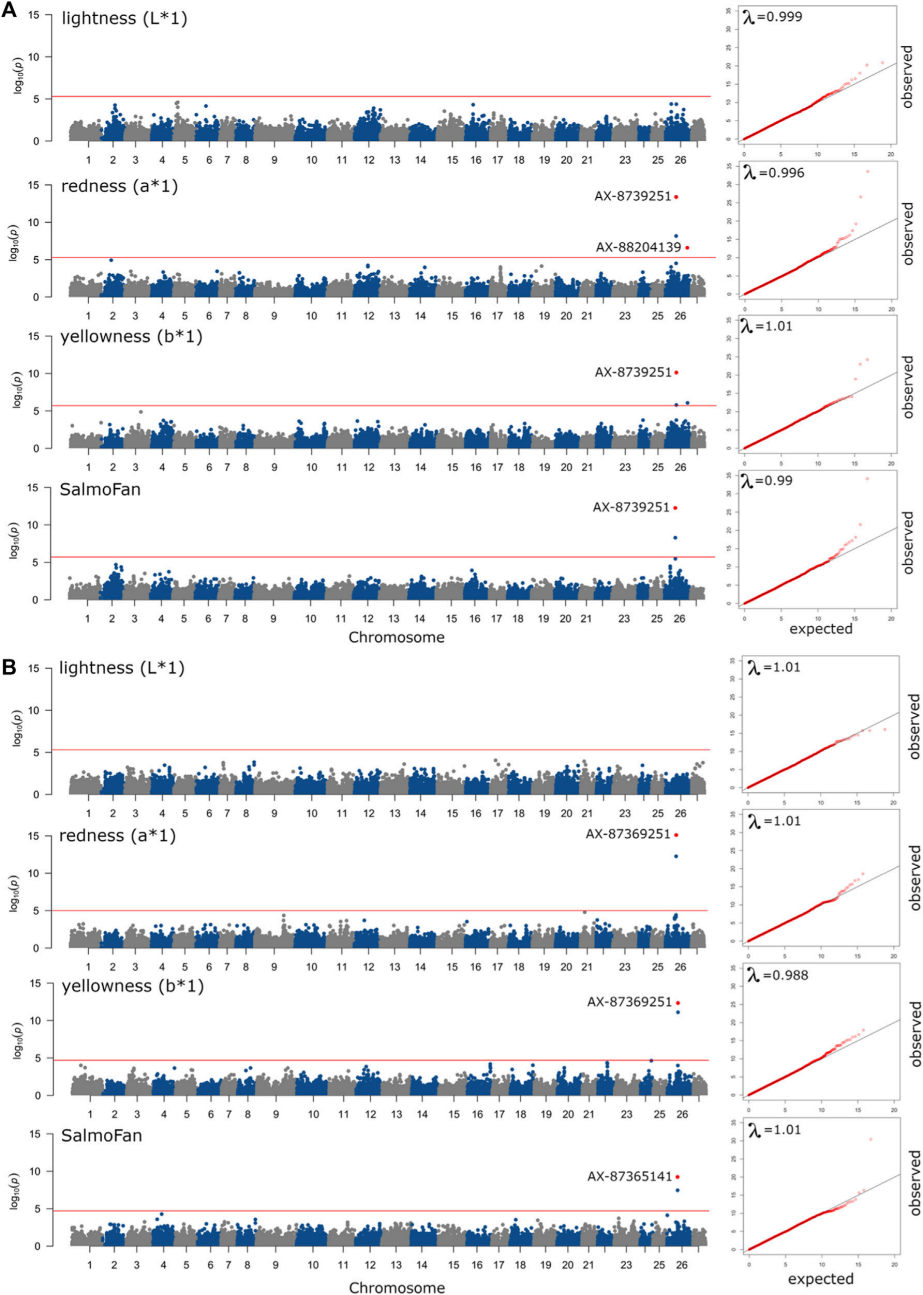
Manhattan plots of ASReml-gwas genome-wide associations from the **(A)** 2018 YC and **(B)** 2019 YC for above the midline measurements of lightness, redness, yellowness, and SalmoFan. The red line is a genome-wide line set to a *p*-value of less than 5% based on the False Discovery Rate (5.0e-6 or 2.0e-6), and the points colored in red with SNP marker labels were found to be significant after correction for the false discovery rate. QQ plots with lambda (λ) values are in cut-outs on the right end of each Manhattan plot, where a λ-value close to 1.00 signifies little to no population stratification in the data.

**FIGURE 5:**
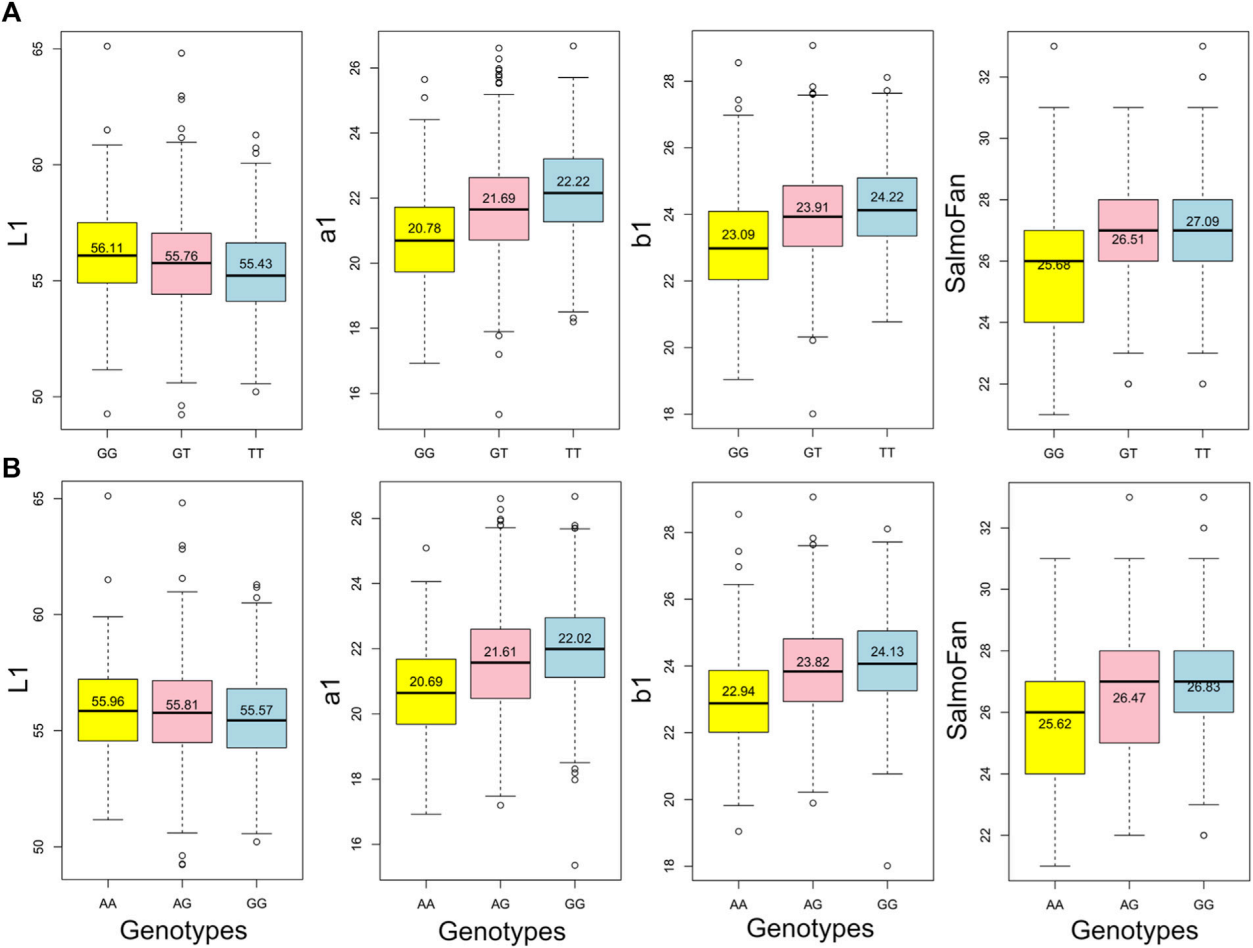
Box plots of the two highly significant SNPs where the effect of alleles can be observed for each of the above midline traits in the 2018 YC, where **(A)** is AX-87369251 and **(B)** is AX-87365141. The values listed on each box are the mean of each genotype.

The results (Manhattan plots, QQ plots, and box plots of major significant SNPS) from the GWAS analysis are presented in Figure 4, Figure 5, and Supplementary Figures S2, S3, respectively. The QQ plots and lambda values (i.e., not significantly different from 1) indicated that possible population stratification was accounted for using the linear mixed-model approach implemented in *ASRgwas*. For both the 2018 and 2019 YC, we found the same consistently elevated genomic region on chromosome 26 associated with a*, b*, and SalmoFan (Figure 4; Supplementary Figure S2). The 2019 YC had less overall noise than the 2018 YC; however, the 2018 YC also displayed the same significant loci at chromosome 26. Using a *p-*value under 5.0e-06 and a false discovery rate under 5%, significant markers were accounted for about 4.08%–14.71% of the additive genetic variation in the color traits (Table 3). Markers AX-87365141 and AX-87369251 were consistently significant for most of the color traits, and they consistently explained the highest percentage of genetic variation (between 6.01% and 12.54%) in redness and yellowness traits (Table 3). Box plots of these two SNPs show the effect on the alleles at these positions, where there is an increase in a*, b*, and SalmoFan traits in certain alleles (Figure 5; Supplementary Figure S3). SalmoFan measurements mirrored redness and yellowness traits more closely in 2018 than 2019 YC (Table 3), which is consistent with higher correlation values in 2018 YC as well (Table 2).

**TABLE 3:** Percentage of genetic variance explained by the top significant markers for all color traits.

Year	Trait	Marker	Exp var^a^
2018	L*1ǂ		(3.26%)
	a*1	AX-87365141, AX-87369251, and AX-88204139	12.51% (9.81%)
	b*1	AX-87369251 and AX-88204139	6.76% (6.96%)
	SalmoFan	AX-87365141 and AX-87369251	9.43%
	L*2	AX-87365141 and AX-87369251	6.30%
	a*2	AX-87365141 and AX-87369251	11.87%
	b*2	AX-87369251	4.08% (6.01%)
2019	L*1ǂ		(0.88%)
	a*1	AX-87365141 and AX-87369251	9.73%
	b*1	AX-87365141 and AX-87369251	8.13%
	SalmoFan	AX-87365141 and AX-87369251	4.30%
	L*2ǂ		(1.46%)
	a*2	AX-520346036, AX-87365141, and AX-87369251	14.71% (12.54%)
	b*2	AX-87365141 and AX-87369251	9.39%

ǂsymbol next to trait indicates there were no significant markers with an FDR below 5%.

^a^ Values in parentheses represent the variance explained by these two markers (AX-87369251 and AX-87365141) if additional/different markers were also significant.

No significant markers were observed for lightness above the midline in the 2018 YC and above and below the midline in the 2019 YC (Figure 4; Supplementary Figure S2). However, for the 2018 YC, there were significant markers (AX-87365141 and AX-87369251) for lightness below the midline. These two markers explained about 6.30% of the genetic variation in lightness below the midline in the 2018 YC.

### 3.4 Gene region discovery

Using a 50 K-base pair (Kbp) buffer on either side of the two SNPs on chromosome 26, Ensembl identified four protein-coding genes that corresponded with the peak: *beta-carotene oxygenase 1* (*bco1*), *host cell factor C1* (*hcfc1*), *boIA family member 3* (*bola3*), and *vesicle amine transport like 1* (*vat1l*) (Table 4). Three additional genes were also found within 50 Kbp of the significant marker on chromosome 21 (position 17,065,604): *rap guanine nucleotide exchange factor 4* (*rapgef4*), *sterile alpha motif and leucine zipper-containing kinase AZK* (*zak*), and *cell division cycle-associated 7a* (*cdca7a*) (Table 4). The final locus on chromosome 26 (position 86,044,190) was not found within or close to a gene. There was no overlapping Gene Ontology terms based on Metascape analysis.

**TABLE 4:** Top SNPs and the associated genes found within 50K base pairs on either side of the SNP.

Trait	SNP	Chrom	Gene (20K)
a*	AX-520346036	21	*rapgef4*, *zak*, and *cdca1a*
SalmoFan	AX-87365141	26	*bco1*, *hcfc1*, *bola3*, and *vat1l*
L*, a*, b*, and SalmoFan	AX-87369251	26	*bco1*, *hcfc1*, *bola3*, and *vat1l*
a* and b*	AX-88204139	26	*-*

*rapgef4* is *rap guanine nucleotide exchange factor 4* (*rapgef4*), *zak* is *sterile alpha motif and leucine zipper-containing kinase AZK*, *cdca1a* is *cell division cycle-associated 7a, bco1* is *beta-carotene oxygenase 1*, *hcfc1* is *host cell factor C1*, *bola3* is *boIA, family member 3*, and *vat1l is vesicle amine transport like 1* (*vat1l*).

## 4 Discussion

The use of genomics in breeding has become an additional tool in understanding trait heritabilities and the genetic architecture of economically important traits. Here, we were able to identify a significant region on chromosome 26 associated with redness, yellowness, and SalmoFan measured fillet color traits, using two YC (both with different parental crosses), of North American-origin Atlantic salmon. The most relevant gene found within this region on chromosome 26 was *bco1*, which is known to be involved in carotenoid metabolism (Helgeland et al., 2019; Blay et al., 2021; Ahmed et al., 2022; Sae-Lim et al., 2022). Lightness occasionally had significant loci in the same region on chromosome 26.

### 4.1 Pedigree and genomic-based heritabilities

We found moderate-to-high pedigree-based heritability in each trait over both years (ranging from 0.36 to 0.55), suggesting that selective breeding can potentially improve the traits. Genomic-based heritability was slightly higher than pedigree-based heritability in the 2018 YC but slightly lower in the 2019 YC; however, all estimates were within the standard error of each other. Similar work on the 2011 YC from the breeding program reported *h* ^2^ values between 0.42 and 0.58 (Garber et al., 2019). The 2011 YC was the grand-parental generation of the 2019 YC, and therefore, the *h* ^2^ estimate was expected to be similar. Previous work on European Atlantic salmon reported heritabilities of a* and b* around 0.20, while the *h* ^2^ estimate of SalmoFan was between 0.12 and 0.14 (Norris and Cunningham, 2004). Recently, Sae-Lim et al. (2022) reported the *h* ^2^ estimate of 0.44 for astaxanthin concentration in the fillet (a measure of redness) of the Mowi population in Norway. Studies on rainbow trout using all three measurements (L*, a*, and b*) reported heritabilities between 0.16 and 0.46 (Blay et al., 2021; Ahmed et al., 2022). Based on the similar heritabilities reported by others, it is highly likely that improvements can be made to future generations by selecting fish with ideal traits.

### 4.2 Beta-carotene oxygenase 1 [like] (bco1 and bco1l) genes in muscle pigmentation

In general, the GWAS does not necessarily identify causal genes but find regions of the genome that are highly linked with causal genes. In both YC and for three of the four traits (a*, b*, and SalmoFan), we found the same strong peak on chromosome 26 that was close to the *beta-carotene oxygenase 1* (*bco1*) gene. Carotenoids are responsible for the muscle coloration in fish such as Atlantic salmon and rainbow trout (Matthews et al., 2006), and *bco1* and *bco1l* are important genes in the variation in carotenoid metabolism in fish. Previous genome-wide association studies on European Atlantic salmon found *bco1* and *bco1l* to be important in red coloration of flesh (Helgeland et al., 2019; Kuhn, 2022; Sae-Lim et al., 2022). Functional experiments further indicated that *bco1l* was more likely involved in redness coloration than *bco1* (Helgeland et al., 2019; Kuhn, 2022). However, mapping of the North American Atlantic salmon genome to the European genome puts the two loci we identified here in the same region as found in Helgeland et al. (2019), which overlaps with the *bco1l* gene.

Despite chromosomal differences and rearrangements between the North American and European Atlantic salmon, we also found *bco1* in high linkage with all our coloration phenotypes. Additionally, the two main loci found in *bco1* were able to explain between 6.01% and 12.54% of the variation found in redness and yellowness phenotypes. Previous work on rainbow trout discovered similar chromosomal peaks with the most significant markers explaining about 2.5%–3.5% of the variation in color phenotypes, of which they identified *beta-carotene 15,15′-dioxygenase* as an important contributor (Ahmed et al., 2022). Given the large amount of variation explained by the two loci on chromosome 26 and the previous work on European Atlantic salmon, *bco1* could be important in the pigmentation of fillets in North American Atlantic salmon.

This is also the first known report of linkage between the *bco1* gene with not only the redness trait in Atlantic salmon but also the yellowness and lightness traits as well. Similar work using all three traits in rainbow trout found the *bco1l* gene important in coloration traits; however, it was specifically found in association with yellowness rather than redness (Blay et al., 2021; Ahmed et al., 2022). It is possible that the blue–yellow coloration index of the yellowness trait is associated with, not caused by, *bco1* or *bco1l*, although functional studies would be able to verify this.

In general, very few markers at the genomic region of interest (chromosome 26 that was discovered in this study to be significant for most of the color traits) crossed the *p*-value/q-value threshold. We suspect that this is because of the density of the marker panel after quality checking the genomic dataset (−33 K markers). It may be prudent to re-evaluate these traits with a larger, more informative marker panel in the future. This is the first version of a North American high-density array, and while the Huntsman breeding program contributed to it and worked with the authors of the array, it may not be the best suited for the specific Saint John population.

### 4.3 Other putative genes and their association with muscle pigment

Six additional genes were identified that may be involved in or closely linked with the pigmentation of Atlantic salmon muscle. *BoIA family member 3* (*bola3*) is an important gene in assembling the mitochondrial iron–sulfur cluster, which is essential for energy metabolism (Uzarska et al., 2016). A study was able to show that knocking out the *bola3* gene resulted in the inhibition of thermogenesis activity and is essential in maintaining mitochondrial homeostasis and adrenergic signaling-induced lipolysis, as shown in Bai et al. (2021). Vesicle amine transport 1 like (VAT1L) is a protein that is predicted to enable oxidoreductase activity and zinc ion-binding activity, and it is a candidate gene identified in obesity and thermogenesis (Chen, 2021).

Host cell factor CI (HCFC1) is a transcriptional protein that is important for regulating cell proliferation, migration, and death (Luciano and Wilson, 2003; Yu et al., 2010). A study of HCFC1 associated this protein with a recessive disorder that results in issues with nervous system and neurological development, difficulty in metabolizing cobalamin (B12), and a general lack of fitness (Yu et al., 2013). A recent zebrafish study was able to show the importance of HCFC1 in craniofacial development (Quintana et al., 2014).

Rap guanine nucleotide exchange factor 4 (RAPGEF4) has been shown to play a role in ion channel function, intracellular calcium signaling, ion transport activity, and exocytosis in somatic cells (Holz et al., 2006). In addition, RAPGEF4 likely mediates the regulation of innate and adaptive immune cell functions (Serezani et al., 2008). Cell division cycle-associated protein 7a (CDCA7A) is a protein that is involved in immune system process, regulation of DNA-templated transcription, cell differentiation, and anatomical structure development (uniport.org). Sterile alpha motif and leucine zipper-containing kinase AZK (*zak*) is a type of mitogen-activated protein kinase kinase kinase (MAPKKK). MAPKKK are signal transduction molecules that have been found to be important in oxidative stress and inflammatory responses (Huang et al., 2009). Dietary carotenoids have been shown to boost disease defense and strengthen resistance to oxidative stress (Nakano and Wiegertjes, 2020). Therefore, based on the functions of all these proteins, it may be possible that muscle color and carotenoids, in general, are highly correlated with pathological responses that may also affect the quality of fillets in Atlantic salmon. Functional studies or genome-wide studies looking into pathogen, metabolism, and deformity traits will help elucidate the connection of the genes identified here to muscle color.

### 4.4 How does SalmoFan compare to the Chroma Meter?

Redness, yellowness, and lightness theoretically make up the coloration of a SalmoFan; therefore, we expected to observe a strong relationship between chromaticity and the SalmoFan. Indeed, there was strong genetic correlations between SalmoFan and a* (redness) and SalmoFan and L* (lightness) (Figure 1; Table 1; Table 2); however, the genetic correlation between SalmoFan and b* (yellowness) was much weaker. Given the weaker correlation of SalmoFan to b*, there may be a need in the future to create a separate metric that combines a*, b*, and L* into a single metric as this would encompass more of the color actually observed in a fillet. For the GWAS, all three traits (a*, b*, and SalmoFan), except lightness, had the same top significant markers on chromosome 26, indicating that these traits may be under pleiotropy genetic control. Due to the lower additive genetic variance and heritability of lightness compared to the other traits, the genetic architecture of lightness may need further investigation with a larger population and phenotype to reveal the true genetic architecture of this trait. Based on genomic results and high correlations, both Chroma Meter and SalmoFan are capable of measuring redness and yellowness traits, and in harvest settings, either could be used depending on what is available.

Interestingly, there was a variation between the years; the 2018 YC had better correlations between SalmoFan and redness/yellowness traits and had a more similar level of explained variation based on chromosome 26 loci (Table 3). This could be due to differences in SalmoFan measurements taken in 2019 (i.e., they may not have been as consistent across the days), but it could also be due, in part, to fillets that were affected by water but not visibly, so therefore, they may not have been included in the group of fillets removed due to the watermark.

## 5 Conclusion

The use of genomics in breeding has become a useful tool in understanding trait heritabilities and the genetic architecture of economically important traits. Here, we were able to identify a specific region on chromosome 26 associated with Chroma Meter traits (redness and yellowness) using two YC of North American Atlantic salmon. We were able to associate redness and yellowness traits specifically with the *bco1* gene, while genetic markers were mostly not significant for the lightness trait. SalmoFan was highly genetically correlated with redness and lightness, but it was moderately correlated with yellowness. Furthermore, the same genomic region identified as significant for the Chroma Meter traits was significant for SalmoFan also, hence, selection for SalmoFan will indirectly improve Chroma Meter traits and *vice versa*. This study provides insights into the genomics of the color traits in the North American Atlantic salmon and will aid in the selection to obtain desired fillet color.

## Data Availability

The original contributions presented in the study are included in the article/Supplementary Material, further inquiries can be directed to the corresponding authors.
