# Supplementary material for: Genome-wide association identifies genomic regions influencing fillet color in Northwest Atlantic salmon (Salmo salar Linnaeus 1758)
Source: Front Genet. 2024 Jul 26;15:1402927. doi: 10.3389/fgene.2024.1402927 (PMC11310022; doi:10.3389/fgene.2024.1402927)
Supplement: Supplementary file 1 [file Table1.DOCX]

Supplementary Table 1: ASReml-R magnitude of effect (i.e., solution matrix table) from fixed effects. Fixed effects were added to the linear trait model for each of the traits of interest, for each year class individually.

| Year | Trait | Intercept | Duration | Day | Hour |
| --- | --- | --- | --- | --- | --- |
| 2018 | L* mean | 47.197 ± 1.337 | 0.006 ± 2.82e-4* | 1: 0.000  2: -0.328 ± 0.104* | -0.268 ± 0.037** |
|  | a* mean | 25.583 ± 1.130 | -0.002 ± 5.72e-4** | 1: 0.000  2: -0.367 ± 0.088* | -0.007 ± 0.031 |
|  | b* mean | 29.879 ± 1.016 | -0.003 ± 5.17e-4** | 1: 0.000  2: -0.768 ± 0.079** | 0.063 ± 0.048* |
|  | SalmoFan | 31.154 ± 1.418 | -0.002 ± 7.21e-4* | 1: 0.000  2: -0.533 ± 0.110* | 0.013 ± 0.039 |
| 2019 | L* mean | 65.706 ± 1.360 | -0.003 ± 4.87e-4** | 1: 0.000  2: -4.346 ± 0.255  5: -2.884 ± 0.187** | -0.118 ± 0.034* |
|  | a* mean | 17.556 ± 1.114 | 9.09e-4 ± 4.10e-4** | 1: 0.000  2: 2.374 ± 0.209  5: 1.843 ± 0.154** | 0.118 ± 0.028 |
|  | b* mean | 20.858 ± 1.110 | 7.61e-4 ± 3.99e-4** | 1: 0.000  2: 1.410 ± 0.208  5: 1.436 ± 0.153** | 0.068 ± 0.028 |
|  | SalmoFan | 27.887 ± 1.505 | -4.35e-4 ± 5.21e-4* | 1: 0.000  2: 0.105 ± 0.282  5: -0.231 ± 0.207 | -0.011 ± 0.038 |

* p-value less than 0.05, ** p-value less than 2.2e-10


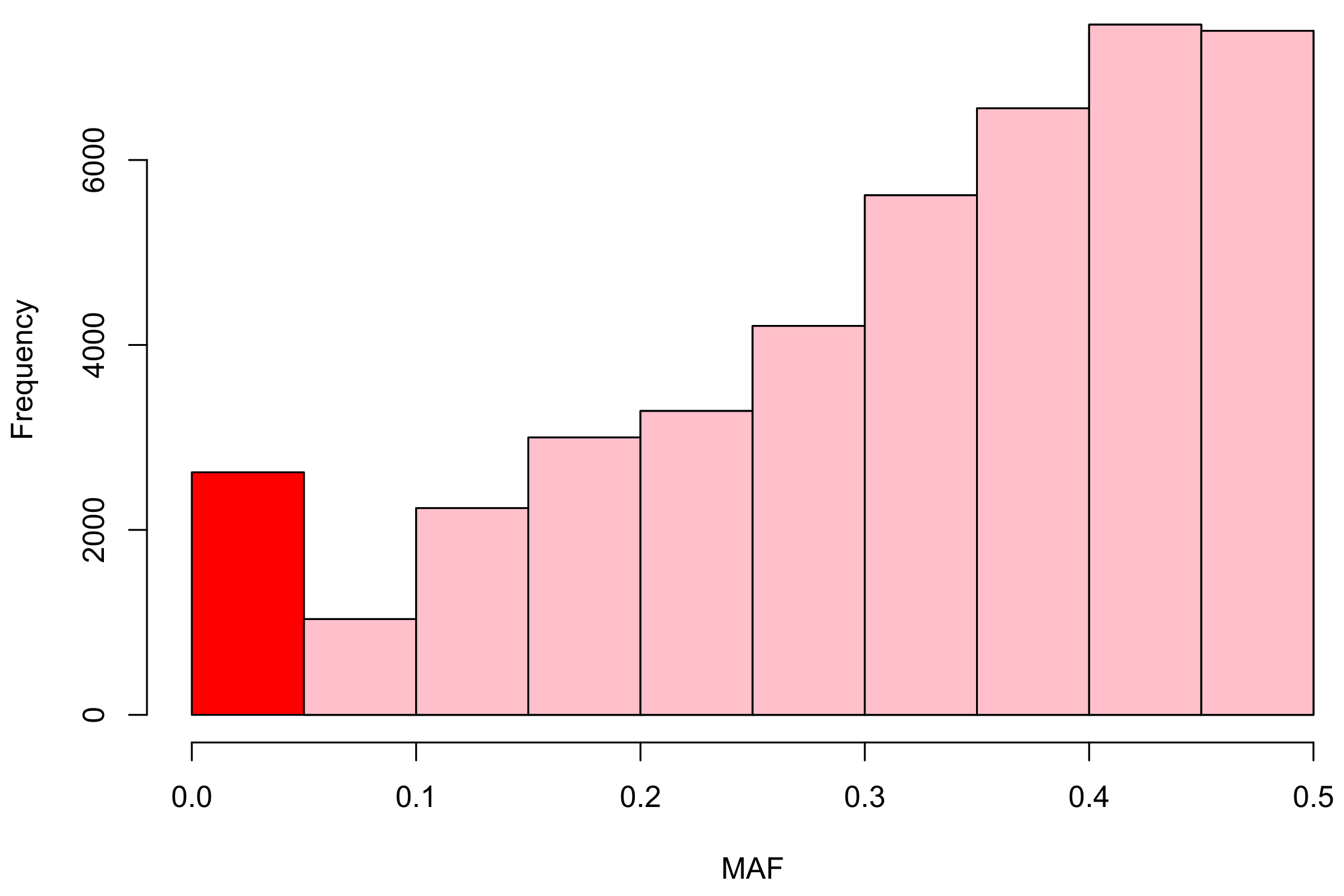
Supplementary Figure 1: Example histogram plot of minor allele frequency (MAF) for the 2018 year class. The pink bars are the SNPs that have been retained before and after filtering for MAF of 0.05 while the bar in red were the SNPs that ended up getting filtered out.


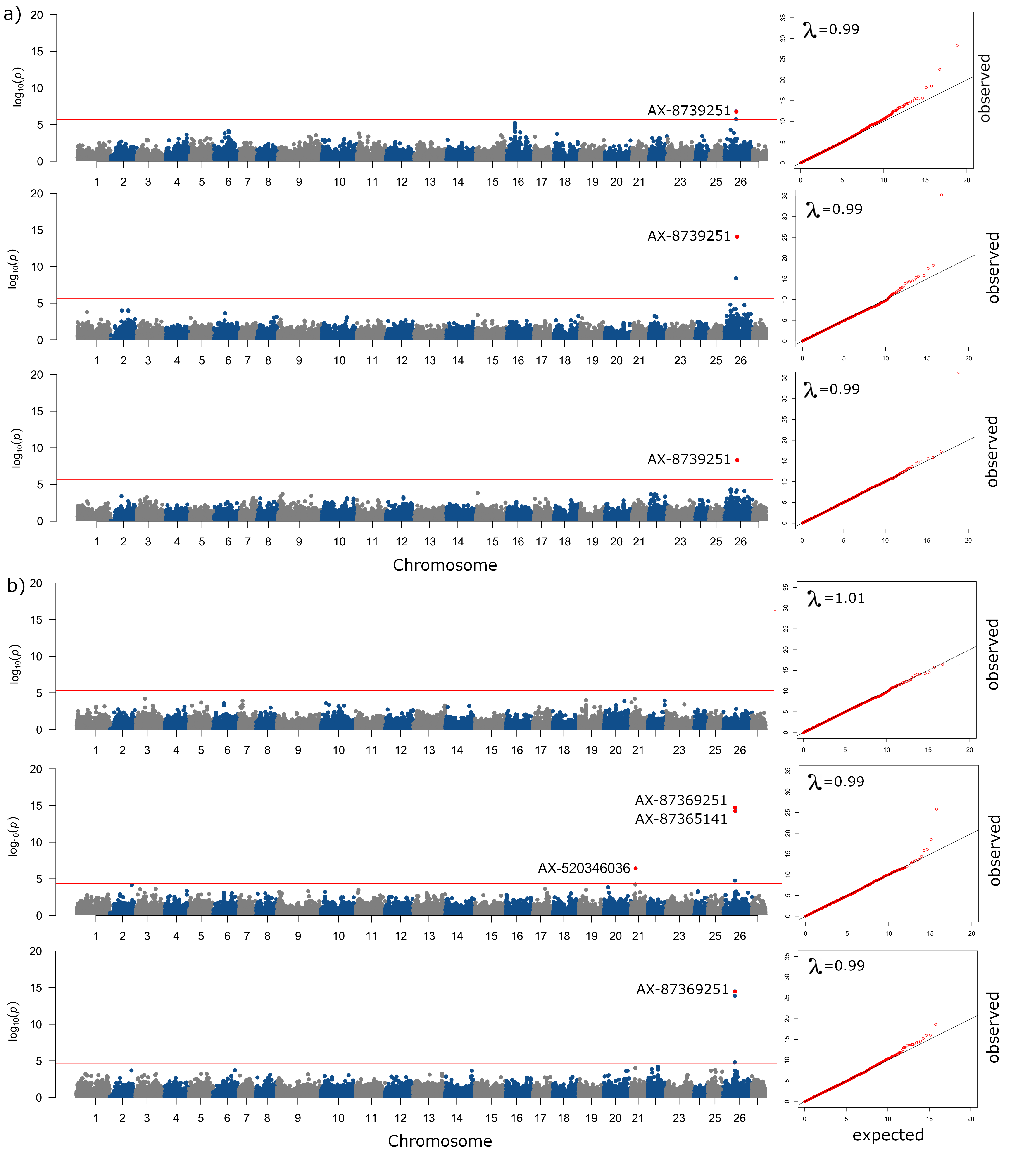


Supplementary Figure 2: Manhattan plots of ASReml-gwas genome wide associations from the a) 2018 and b) 2019 year class (YC) for **below midline** measurements of: lightness, redness, and yellowness, respectively. The red line is a genomewide line set to the *p*value from Table 3 and the points colored in red with SNP marker labels were found to be significant after correction for false discovery rate and backwards selection. QQplots with lambda (λ) values are in cut-outs on the right end of each Manhattan plot, where a λ value close to 1.00 signifies little to no population stratification in the data.


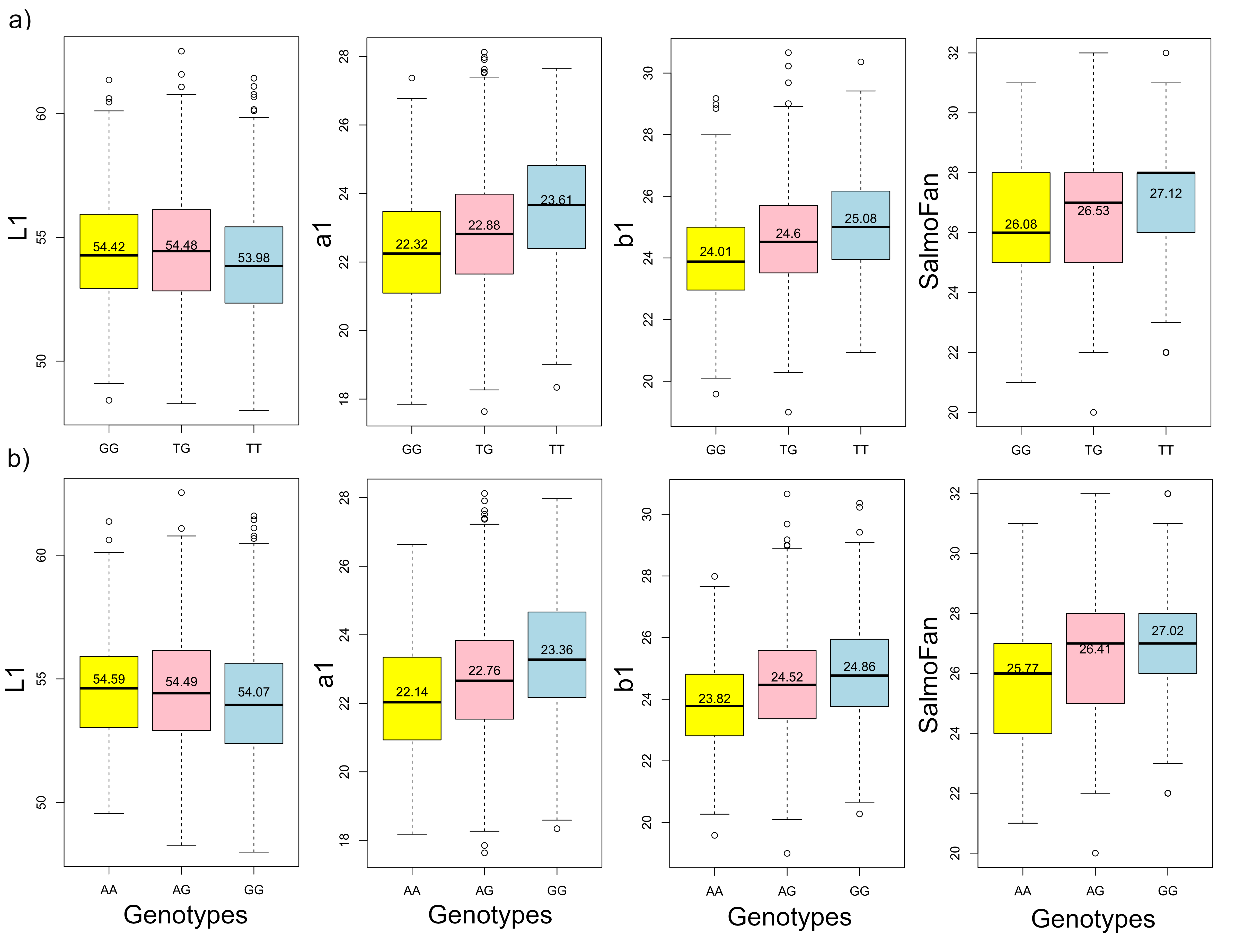


Supplementary Figure 3: Boxplots of the two highly significant SNPs where the effect of alleles can be observed for each of the **above midline** traits in the 2019 year class, where a) is AX-87369251 and b) is AX-87365141. The values listed on each box is the mean of each genotype.
